# Reducción de costos en salud por obras de agua y alcantarillado en Buenos Aires

**DOI:** 10.26633/RPSP.2019.27

**Published:** 2019-03-20

**Authors:** María Victoria Favilla, Mariana Conte Grand

**Affiliations:** 1 Agua y Saneamientos Argentinos Agua y Saneamientos Argentinos Buenos Aires Argentina Agua y Saneamientos Argentinos, Buenos Aires, Argentina.; 2 Universidad del Centro de Estudios Macroeconómicos de Argentina (UCEMA) Universidad del Centro de Estudios Macroeconómicos de Argentina (UCEMA) Buenos Aires Argentina Universidad del Centro de Estudios Macroeconómicos de Argentina (UCEMA), Buenos Aires, Argentina.

**Keywords:** Salud ambiental, años potenciales de vida perdidos, costo de enfermedad, medición de riesgo, Argentina, Environmental health, potential years of life lost, cost of illness, risk assessment, Argentina, Saúde ambiental, anos potenciais de vida perdidos, custos da doença, medição de risco, Argentina

## Abstract

**Objetivo.:**

Estimar el beneficio que reportaría la reducción en la cantidad de casos de diarrea generada por la mejora de las condiciones de agua, saneamiento e higiene de la población bajo estudio (25 partidos en Buenos Aires).

**Métodos.:**

La estimación se realiza en términos de ahorro de años de vida ajustados por discapacidad, a los cuales se les asigna un valor monetario. Sobre esta base se hace un análisis probabilístico considerando la incertidumbre de 15 variables. También se considera la posible subestimación de los datos de salud.

**Resultados.:**

Los beneficios en salud de alcanzar un acceso ideal de agua y saneamiento en el área cubierta ascienden a 3 262,0 millones de pesos argentinos (ARS), equivalentes a 203,9 millones de dólares estadounidenses (USD). Estos varían entre USD 67,5 millones y 559,5 millones una vez realizado el análisis de sensibilidad.

**Conclusiones.:**

Los beneficios calculados (ahorro de costos en salud por diarreas) ajustados por subestimación de datos de salud, cubrirían 35,3% de los costos de inversión, considerando una cobertura cercana a 95% en agua y a 85% en saneamiento. Por el alcance limitado de este trabajo, los beneficios calculados constituyen un límite inferior. Posibles extensiones serían considerar las reducciones de costos por otras enfermedades, ahorros de tiempo e impactos en el medio ambiente.

## Abstract (en)

**Objective.:**

To estimate the benefits that would be obtained by reducing the number of diarrhea cases through improved water, sanitation, and hygiene conditions in the study population (25 districts in Buenos Aires).

**Methods.:**

The benefits were estimated by calculating the number of disability-adjusted life years saved, which were given a monetary value. On this basis, a probabilistic sensitivity analysis was carried out, considering the uncertainty in 15 variables. We also considered potential underestimation of the health data.

**Results.:**

The health benefits of improved access to water and sanitation in the area covered amounted to 3.262 billion Argentine pesos (ARS), equivalent to 203.9 million US dollars (USD), with a range between USD 67.5 and 559.5 million, based on the sensitivity analysis.

**Conclusions.:**

The calculated benefits (savings in diarrhea-related health costs), adjusted for underestimation in the health data, would cover 35.3% of the investment costs, with around 95% coverage for water and 85% for sanitation. Since this research is limited in scope, the calculated benefits represent a minimum level. Further research could consider cost reductions for other diseases, time savings, and environmental impact.

## RESUMO

**Objetivo.:**

Estimar o benefício que traria a redução no número de casos de diarreia decorrente de melhoria nas condições de água, saneamento e higiene da população estudada em 25 subdivisões administrativas em Buenos Aires.

**Métodos.:**

A estimativa é feita em termos de poupar anos de vida ajustados por incapacidade, aos quais se atribui uma cifra monetária. Uma análise probabilística é feita sobre esta base considerando a incerteza de 15 variáveis. Também é considerada a possível subestimação dos dados de saúde.

**Resultados.:**

Os benefícios em saúde com o acesso ideal à água e saneamento na área estudada chegam a 3 milhões e 262 mil pesos argentinos, um valor equivalente a 203,9 milhões de dólares dos Estados Unidos. Estes valores variam entre 67,5 e 559,5 milhões de dólares após ser realizada a análise de sensibilidade.

**Conclusões.:**

Os benefícios estimados (economia de custos em saúde por diarreia), ajustados para a subestimação de dados de saúde, cobririam 35,3% dos custos de investimento, considerando uma cobertura próxima a 95% em água e 85% em saneamento. Dado o alcance limitado deste estudo, os benefícios estimados representam um limite inferior. Outros possíveis estudos derivados poderiam considerar a redução de custos por outras doenças, economia de tempo e impacto ao meio ambiente.

En Buenos Aires, las epidemias de cólera y fiebre amarilla de 1870 fueron clave para la ampliación del sistema de agua potable y obras de alcantarillado (1). Uno de los últimos trabajos sobre el impacto en salud de las intervenciones de agua, saneamiento e higiene (ASH) concluye que las muertes atribuidas a condiciones inadecuadas de ASH decrecieron de 2,2 millones en 2005 a 1,8 millones en 2015 a nivel global (18,9%). Los años de vida perdidos debido a muerte prematura y a discapacidad (AVAD) ocasionados por las diarreas disminuyeron 26,2% en el mismo período (2), en parte por las mejoras de ASH. Aun así, la mortalidad por diarrea es todavía la novena causa de muerte en el mundo (en 1990 era la quinta causa).

Este trabajo tiene como objetivo cuantificar los beneficios generados por la mejora de las condiciones de ASH, al disminuir la incidencia de la diarrea en la población de 25 partidos de la de Buenos Aires (PBA).^1^

## MATERIALES y MÉTODOS

En el área considerada viven 9,8 millones de personas, que corresponden a 24,5% de la población del país y 62,9% de la población de la PBA. El beneficio se estima a través de los años de vida perdidos debido a muerte prematura y a discapacidad (AVAD) evitados por la mejora de las condiciones de agua, saneamiento e higiene (ASH). La cuantificación de los beneficios asociados a ASH inadecuada no existe en Argentina (excepto por [3]).

### Riesgo atribuible a deficiencias en la provisión de agua y saneamiento

El riesgo atribuible proporcional poblacional (RAPP) es la proporción de casos de diarrea en la población (muertes, hospitalizaciones y casos adicionales) que pueden ser atribuidos a condiciones inadecuadas de ASH. Se calcula con la fórmula [1] (4-6).

$$RAPP=\frac{\sum_{i}pi\cdot RRi-\sum_{i}pi´\cdot RRi}{\sum_{i}pi\cdot RRi}i=\text{ escenario}$$ [1]

Donde:

pi: proporción de la población en cada uno de los escenarios de exposición i.

pi′: proporción de la población en cada uno de los escenarios de exposición i después de hacer ciertas obras.

RRi: riesgo relativo asociado a cada escenario de exposición i comparado con el nivel de referencia.

RR: tasa de enfermedad entre los individuos expuestos, dividido la tasa de enfermedad en los individuos no expuestos: si es > 1 representa una mayor RR.

RR es la tasa de enfermedad entre los individuos expuestos, dividido la tasa de enfermedad en los individuos no expuestos: si es mayor que uno representa una mayor probabilidad de enfermarse en presencia del factor de riesgo.

Para la definición de escenarios se utiliza la metodología de Global Burden of Disease (GBD) 2000-2004 (6). No se adoptan versiones más recientes del GBD debido a que han sido objeto de algunas críticas (7).

De acuerdo al Programa Conjunto de Monitoreo del Abastecimiento de Agua y el Saneamiento de la Organización Mundial de la Salud (OMS) y el Fondo de las Naciones Unidas para la Infancia (UNICEF, por sus siglas en inglés) (8), una fuente de agua mejorada es segura si por su diseño y construcción tiene el potencial de distribuir agua segura, accesible y libre de contaminantes. Una facilidad sanitaria mejorada y de manejo seguro es aquella que separa, de manera higiénica, las heces del contacto humano, no es compartida por otros hogares y puede ser transportada o tratada en forma correcta.

En base a esta definición, y a la información censal disponible, las facilidades como no mejoradas se clasifican en:

- **Saneamiento:** cuando el desagüe sea con descarga de agua a pozo ciego, hoyo u excavación, no tenga descarga de agua, o sea sin baño o letrina.
- **Agua para el consumo:** cuando no provenga de red pública o la cañería no sea dentro de la vivienda con bomba a motor.

Así, la población se distribuye en los siguientes escenarios:

- **Escenario I:** ausencia de transmisión de diarrea por falta de obras de infraestructura de agua y alcantarillado (mínimo riesgo teórico).
- **Escenario II:** oferta por red pública de agua con calidad regulada y tratamiento parcial de efluentes sanitarios en un país con más de 98% de la población cubierta.
- **Escenario III:** conexión a red pública de agua dentro de la vivienda y desagüe sanitario con descarga de agua a red pública en un país con menos de 98% de la población cubierta. Este escenario se subdivide en a y b. Esta subdivisión no los hace independiente uno del otro, sino que es para captar la reducción del riesgo de agua y desagüe sanitario a la red.
- **Escenario IV:** agua y saneamiento mejorados (agua por red pública dentro o fuera de la vivienda o cañería dentro de la vivienda proveniente de una perforación con bomba a motor, y servicio sanitario con desagüe a cámara séptica con descarga de agua, o agua por red pública fuera de la vivienda y facilidad sanitaria con desagüe a red pública o cámara séptica con descarga de agua).
- **Escenario Va**: provisión de agua no mejorada y facilidad sanitaria mejorada (desagüe a red pública o cámara séptica y pozo, con descarga de agua).
- **Escenario Vb**: facilidad sanitaria no mejorada, y mejorada de agua (proveniente de red pública o perforación con bomba a motor con cañería dentro de la vivienda).
- **Escenario VI**: facilidad no mejorada, tanto de agua como de saneamiento.

A medida que se pasa del escenario I al VI, la probabilidad de enfermarse aumenta. En varios de los estudios del GBD no se hace diferencia entre una facilidad sanitaria mejorada (por ejemplo, descarga a pozo) y un servicio con conexión de efluentes sanitarios a la red pública. Aquí se considera en el escenario III a la población con acceso a la red de alcantarillado y a la red de agua potable (no en el IV), porque aún existiría una mejora asociada a tener un sistema de agua y saneamiento seguro (9). Los beneficios se calculan teniendo en cuenta que la población pasa al escenario I o II en el área considerada.

Una vez clasificada la población en los distintos escenarios, se obtiene el grado de exposición diferencial en cada partido según el último Censo de Argentina (2010).

Los RR asociados a condiciones inadecuadas de ASH para la transición entre cada escenario de exposición surgen de la literatura (6, 10). En este trabajo se consideran valores mínimos, medios y máximos, según la disponibilidad de estudios (cuadro 1). Por ejemplo, para la transición entre los escenarios IV y IIIb, se utiliza en el caso base la reducción del riesgo asociada a realizar tratamiento del agua en el lugar de uso con almacenamiento seguro (5, 6, 11): 44,7% (RR = 100/ [100-44,7] = 1,81). Para el límite inferior, se considera la reducción del riesgo de enfermarse al realizar intervenciones sobre la calidad del agua, como tratamiento en el lugar de uso (cloración, hervor, etc.) o protección de la fuente (12) de 39,3% (RR = 1,65). Para el límite superior se utiliza una reducción del riesgo de 46,0% (RR = 1,85), obtenida de una metarregresión de 32 estudios de intervenciones de calidad de agua (13).

**CUADRO 1:** Riesgo para cada escenario de exposición

Conceptos de riesgo	Escenarios													
	I	II^a^	III						IV			V		VI^a^
			IIIa			IIIb						Va^a^	Vb^a^	
			Mín.	Base	Máx.	Mín.	Base	Máx.	Mín.	Base	Máx.	Base	Base	Base
Reducción del riesgo (%)^b^	NA	60,00	33,70	35,00	40,00	31,00	32,00	34,00	39,30	44,70	46,00	16,70	0,00	20,80
Riesgo relativo parcial^b^	NA	2,50	1,51	1,54	1,67	1,45	1,47	1,52	1,65	1,81	1,85	1,20	1,00	1,26
Riesgo relativo absoluto^c^	1,00	2,50	3,77	3,85	4,17	5,46	5,66	6,31	9,00	10,23	11,69	12,28	12,28	15,50
Riesgo relativo absoluto^d^	NA	1,00	1,51	1,54	1,67	2,19	2,26	2,53	3,60	4,09	4,68	4,91	4,91	6,20

Cuadro de elaboración propia con base en (9).

a Para estos escenarios se toma un solo valor ya que no existen riesgos relativos validados para niveles bajo y medio.

b De cada escenario respecto al escenario anterior.

c De cada escenario respecto al escenario I.

d De cada escenario respecto al escenario II.

NA, no aplicable.

### Años de vida ajustados por discapacidad (AVAD)

Para estimar los casos de diarrea atribuidos a las malas condiciones de ASH, se multiplica la fracción atribuible calculada de acuerdo a la ecuación [1] por la cantidad de casos de diarrea estimados en términos de AVAD:

Mortalidad o morbilidad atribuida a diarrea = RAPP x AVAD [2]

Donde:

RAPP: riesgo atribuible proporcional poblacional.

AVAD: suma de los años de vida perdidos por muerte prematura (AVMP) y los años de vida perdidos por condiciones de discapacidad (AVD).

Se entiende por discapacidad a cualquier condición de salud imperfecta: problemas de movilidad, incapacidad de participación en actividades usuales, dolor o incomodidad constantes, ansiedad, depresión o deterioro cognitivo.

El cálculo de AVAD surge de (14):

$$\begin{array}{l} AVAD=D.[\frac{K.C.e^{r.a}}{{\left(r+\beta \right)}^{2}}\;.[e^{-\left(r+\beta \right)\left(L+a\right)}.\;\left(-\left(r+\beta \right).\left(L+a\right)-1\right)-\\ \begin{array}{cc} & e^{-\left(r+\beta \right).a}.\;\left(\;-\left(r+\beta \right).\;a-1\right)]+\frac{1-K}{r}\;.\;\left(1-e^{-rL}\right)] \end{array} \end{array}$$ [3]

Donde:

D: constante de peso o gravedad de la enfermedad.

K: constante de moderación del peso de la edad.

C: constante de corrección del peso de la edad.

B: constante de peso de la edad.

r: tasa de descuento.

a: edad del paciente.

L: expectativa de vida (AVMP) o duración de la enfermedad (AVD).

Para calcular los AVAD se parte del número de muertes ocurridas por diarrea y el número de casos de enfermedad, tanto las que requirieron hospitalización como las que no. Se consideran las enfermedades infecciosas intestinales (A00 a A09), tracoma (A71) y enfermedades parasitarias (B65 a B83) incluidas en la Clasificación Internacional de Enfermedades 10° edición (CIE-10), tal como en la literatura relacionada (6). Los datos de egresos hospitalarios del subsector público oficial y defunciones fueron provistos por la Dirección de Estadísticas e Información en Salud del Ministerio de Salud de la Nación y las notificaciones (eventos de diarrea registrados que no resultan en internaciones), por la Dirección de Epidemiología del Ministerio de Salud de la PBA. En los datos se presentan casos de varios de los códigos requeridos, aunque no de todos.

Para **L** en AVMP, se utiliza la Tabla abreviada de expectativa de vida por grupo de edad para ambos sexos publicada por el Instituto Nacional de Estadísticas y Censos (INDEC) para la PBA (15). Para **L** en AVD, se toma la estadía media en establecimientos municipales por casos de hospitalizaciones de diarrea: 2,71 días para el año 2013 (16). Dado que el diagnóstico puede no ocurrir en forma inmediata, y que una vez que el paciente sale del hospital puede no encontrarse en condiciones de retomar sus actividades, se considera **L** = 7 días (mínimo: 4 días; máximo: 14 días) para los egresos hospitalarios. Por otra parte, se considera **L** = 3 días (mínimo: 2 días; máximo: 4 días) para las notificaciones.

Para **D**, GBD usa una encuesta en la que se comparan dos estados de salud descritos en términos de efectos funcionales y síntomas a que se ven sometidos los individuos hipotéticos y el encuestado debe decidir qué estado es más saludable (17, 18). Dadas las críticas que reciben este tipo de encuestas (19, 20), se toman valores alternativos. Para el escenario base se utilizan los **D** estimados para el GBD 2013, los cuales se estiman con información de varios países como para capturar las perspectivas de diferentes comunidades (17). Para egresos hospitalarios, se considera **D** = 0,247 (diarreas graves) y para notificaciones, **D** = 0,188 (gravedad media). Los **D** estimados por (20) y (18) se utilizan como valores mínimos y máximos, respectivamente.

La idea para incorporar las constantes de peso de la edad es que habría una preferencia por la sociedad a valorar más un año de vida vivido por un adulto joven que por un anciano o un niño (14), ya que el adulto es más productivo. Para ponderar la edad **a** en el cálculo de los AVAD se incluye K.C.a.e ^−βa^ en [3]. Si **K** = 0, la edad no pesa, mientras que si **K** = 1, la edad pesa completamente y depende de los parámetros **β** y **C** (21). No todos los estudios concuerdan que a los jóvenes y a los adultos mayores haya que asignarles menor peso; por eso, para el escenario base se considera **K** = 0 y para el análisis de sensibilidad se permite **K** = 1. El valor medio de β que suele utilizarse es 0,04 (4), que implica un máximo de ponderación a los 25 años de edad. Los valores mínimo (0,02) y máximo (0,06), escogidos siguiendo a los autores del GBD (21), implican una ponderación máxima a la edad de 50 y 17 años, respectivamente.

La tasa de descuento (r) incorpora una preferencia intertemporal: cuanto mayor es, menor resulta el valor que el individuo le asigna a su salud en el futuro que en el presente. Se utiliza para el escenario base una tasa de 3% (4) y para el análisis de sensibilidad un valor mínimo de 1% y máximo de 5% (22).

### Valor actual de los beneficios por reducción de la diarrea

Luego de obtenidas la mortalidad y la morbilidad evitadas en términos de AVAD, los convertimos en dinero multiplicándolos por el ingreso. Se supone una evolución de los AVAD de acuerdo al crecimiento poblacional para cada partido, proyectado por el Instituto Nacional de Estadísticas y Censos (INDEC) en base al Censo 2010 (23).

El valor presente de los beneficios se obtiene con la ecuación:

$$VP=\sum_{t=0}^{T}\frac{B_{t}}{{\left(1+d\right)}^{t}}$$ [4]

Donde:

Bt: beneficio en cada período t (AVAD evitados por ingreso).

d: tasa de descuento.

t: período de tiempo.

T: horizonte de valuación.

Como las enfermedades parasitarias e infecciosas se transmiten sobre todo en contextos con inadecuadas condiciones de ASH, se supone que la mayor parte de las personas afectadas corresponden a grupos ingresos bajos. Por ello, se considera para el escenario de mínima, el ingreso mínimo, vital y móvil (24) para 2016 (ARS 6 748/mes, que equivalían a USD 422 en 2016).^4^ Para el escenario de máxima se toma (25) la remuneración promedio del año 2016 en el sector privado (ARS 17 742/mes, que equivalía a USD 1 109).

Se considera un horizonte temporal de 40 años para las conexiones domiciliarias de agua y alcantarillado (26), se utilizaron límites de 30 y 50 años para el análisis de sensibilidad.

No existe consenso en la literatura sobre la mejor manera de descontar flujos de fondos derivados de proyectos sociales (27). Se utiliza la tasa calculada para Chile (5,05%) en el caso base, para Argentina (2,36%) como mínimo (28) y, como máximo, 12%, recomendado por los organismos multilaterales de crédito.

Para el análisis de sensibilidad se utiliza Simula 4.0^®^ (29) con distribuciones triangulares (excepto para **K**, que se supone una binomial).

## RESULTADOS

Del análisis de los datos censales se desprende el porcentaje de población que se encuentra en cada escenario (cuadro 2). La disparidad entre partidos es amplia. Al considerar el conjunto del área, 32,4% de la población se encuentra en el escenario III, 30,3% de la población tiene acceso a servicios mejorados de agua y alcantarillado (escenario IV) y el 27,9% tiene acceso a servicio de agua mejorado pero no de saneamiento, lo que confirma la tendencia mundial que la provisión de saneamiento va detrás de la de agua mejorada (2). Con los RR (cuadro 1) y la población en cada escenario (cuadro 2), se calcula el RAPP en el área bajo estudio: en el escenario I podrían evitarse en promedio 89,5% de los casos de diarrea (y 73,7% de los casos, para pasar al escenario II), en línea con estimaciones internacionales 85-90% para países en desarrollo (6).

La cantidad de defunciones por diarrea para el período 2001-2014 en el área bajo estudio es de 53,2 en promedio por año (59 en 2013): 45,5% ocurre en niños menores de un año y 40,7% en adultos mayores de 70 años. En cuanto a los egresos hospitalarios, los mismos son en promedio, 4 136 por año (5 062 en 2013) para el período que se dispone de datos (2005-2013): 54% ocurre en niños menores de 5 años y 10,9% en adultos mayores a 70 años. Las notificaciones para el período del que se tienen datos (2006-2015) son, en promedio, 132 637 por año (135 108 en 2013).

Es de esperar que, como en otros países, los datos de salud estén subestimados (30). La mortalidad se suele reportar como “paro cardiorrespiratorio no traumático” aunque el origen haya sido una deshidratación por diarrea. En las notificaciones, es posible que la diarrea no se reporte por no considerarla importante y puede haber subestimación si las personas no buscan ayuda médica (31). Por estos motivos, se opta por estimar un sesgo “bajo” (casos 3,3 veces mayores a los registrados) (31) y uno “alto” (10 veces más casos que los registrados) (30). En el caso de nuestros datos, existe un sesgo adicional ya que los egresos hospitalarios corresponden solo a los hospitales públicos.

En el cuadro 3 se resumen, como ejemplo, los eventos de salud reportados en el año 2013 (año en que hay datos completos de los tres tipos de eventos), los casos y los AVAD evitados al pasar al escenario I. Las defunciones evitadas para el caso base serían 52,9 y los AVAD se reducirían en 1 053,2. Para los egresos hospitalarios y las notificaciones, los AVAD resultan inferiores a los casos registrados. Esto se debe a que una diarrea implica pocos días de enfermedad y a que su gravedad es baja. Al pasar al escenario I se evitarían 21,6 AVAD por 4 555 egresos hospitalarios evitados y 187,0 AVAD por 120 685,9 notificaciones evitadas. Las defunciones evitadas dan cuenta de 83,5% de las AVAD, mientras las notificaciones evitadas representan 14,8%.

**FIGURA 1:**
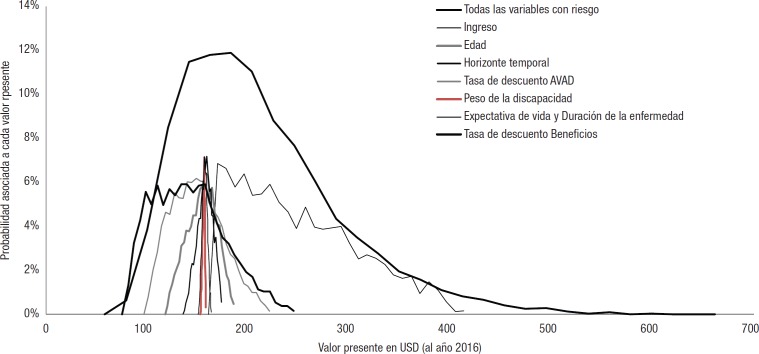
Distribución de probabilidad del valor a 2016 de los beneficios proyectados: toda la población con cobertura ideal de agua y alcantarillado (escenario I)

En el cuadro 4 se expone el valor presente de los beneficios como resultado del análisis de sensibilidad, que es de tipo probabilístico (una simulación de Monte Carlo con 10 000 iteraciones, considerando la incertidumbre en quince variables). Los beneficios en salud por pasar al escenario I y II ascienden a ARS 3 262,0 millones (USD 203,9 millones) y a ARS 2 701,2 millones (USD 168,8 millones), respectivamente. Los primeros varían entre 67,5 y 559,5 y los segundos, entre 55,7 y 466,0 millones de dólares. Como se observa en la figura 1, al correr simulaciones mediante la fijación de las variables en los valores determinísticos con excepción de una variable por vez, queda claro que el salario y la tasa de descuento son los que más incertidumbre aportan al cálculo de los beneficios evitados.

**CUADRO 2:** Distribución de la población en escenarios

Partidos de la PBA	Escenarios						
	I	II	III	IV	Va	Vb	VI
	Porcentaje de la población (%)						
Almirante Brown	0,0	0,0	12,7	41,0	31,4	2,6	12,4
Avellaneda	0,0	0,0	62,0	15,4	22,2	0,2	0,2
Escobar	0,0	0,0	11,1	39,2	27,7	3,6	18,3
Esteban Echeverría	0,0	0,0	11,7	42,0	33,2	2,6	10,5
Ezeiza	0,0	0,0	3,6	44,1	22,4	4,6	25,3
Florencio Varela	0,0	0,0	21,2	22,2	46,5	1,1	9,1
General San Martín	0,0	0,0	47,5	24,6	27,2	0,2	0,6
Hurlingham	0,0	0,0	7,8	62,6	20,4	2,8	6,4
Ituzaingó	0,0	0,0	5,2	69,3	16,1	3,1	6,4
José C. Paz	0,0	0,0	5,1	43,6	27,8	3,5	20,0
La Matanza	0,0	0,0	37,7	25,4	24,9	2,1	9,9
Lanús	0,0	0,0	33,6	27,9	38,3	0,1	0,1
Lomas de Zamora	0,0	0,0	25,3	28,9	44,7	0,2	0,9
Malvinas Argentinas	0,0	0,0	1,2	51,2	28,7	3,2	15,7
Merlo	0,0	0,0	13,3	41,2	29,3	2,6	13,6
Moreno	0,0	0,0	12,8	37,1	30,0	2,9	17,2
Morón	0,0	0,0	49,1	34,6	12,9	1,1	2,3
Presidente Perón	0,0	0,0	1,2	39,5	41,6	1,8	15,9
Quilmes	0,0	0,0	51,3	13,0	35,2	0,2	0,4
San Fernando	0,0	0,0	70,2	9,1	18,8	0,6	1,4
San Isidro	0,0	0,0	77,2	12,3	10,1	0,3	0,1
San Miguel	0,0	0,0	17,0	44,9	25,7	2,8	9,6
Tigre	0,0	0,0	15,0	40,9	35,3	2,4	6,4
Tres de Febrero	0,0	0,0	76,8	10,7	10,3	0,6	1,6
Vicente López	0,0	0,0	95,1	1,7	3,1	0,0	0,1
**Total**	**0,0**	**0,0**	**32,4**	**30,3**	**27,9**	**1,7**	**7,7**

PBA, provincia de Buenos Aires.

Elaboración propia.

***Nota:*** la línea más gruesa se refiere a la situación en la que los 15 parámetros se asumen con riesgo. Para el resto de las líneas, la leyenda se refiere a los parámetros cuyo riesgo se considera para la simulación (el resto permanece con el valor de base).

**CUADRO 3:** Eventos de salud, casos y AVAD evitados en 2013, caso base, escenario I

Partidos de la PBA	Casos			Casos evitados			AVAD evitados			
	Defunciones	Egresos hospitalarios	Notificaciones	Defunciones	Egresos hospitalarios	Notificaciones	Defunciones	Egresos hospitalarios	Notificaciones	Total
Almirante Brown	5	114	1 264	4,5	103,6	1 149,1	91,7	0,5	1,8	93,9
Avellaneda	2	45	6 844	1,7	39,3	5 973,7	52,1	0,2	9,3	61,5
Escobar	3	90	11 952	2,7	82,1	10 897	43,3	0,4	16,9	60,5
Esteban Echeverría	3	51	1 478	2,7	46,4	1 343,4	77,2	0,2	2,1	79,5
Ezeiza	2	38	6 644	1,8	34,8	6 088,3	54,7	0,2	9,4	64,3
Florencio Varela	1	63	2 046	0,9	57,1	1 855,1	27,0	0,3	2,9	30,2
General San Martín	3	223	7 853	2,7	197,2	6 945,2	38,2	0,9	10,8	49,9
Hurlingham	1	139	399	0,9	126,0	361,6	8,2	0,6	0,6	9,3
Ituzaingó	0	8	863	0,0	7,3	782,5	0,0	0,0	1,2	1,2
José C. Paz	6	226	4 251	5,5	206,7	3 887,4	127,6	1,0	6,0	134,6
La Matanza	8	339	15 132	7,2	303,6	13 552,7	158,0	1,4	21,0	180,4
Lanús	0	124	10 346	0,0	110,9	9 255,3	0,0	0,5	14,3	14,9
Lomas de Zamora	3	151	6 252	2,7	136,0	5 629,3	38,9	0,6	8,7	48,2
Malvinas Argentinas	1	920	3 486	0,9	841,1	3 187	8,2	4,0	4,9	17,2
Merlo	3	1 418	6 766	2,7	1 289	6 150,5	57,4	6,1	9,5	73,0
Moreno	3	107	2 289	2,7	97,5	2 085	21,0	0,5	3,2	24,6
Morón	0	52	8 719	0,0	45,8	7 680,1	0,0	0,2	11,9	12,1
Presidente Perón	0	8	811	0,0	7,3	742,8	0,0	0,0	1,2	1,2
Quilmes	2	62	7 409	1,8	54,8	6 550,6	40,0	0,3	10,1	50,4
San Fernando	1	99	242	0,9	85,8	209,7	25,9	0,4	0,0	26,6
San Isidro	3	232	13 563	2,6	198,5	11 602,7	28,6	0,9	18,0	47,5
San Miguel	1	132	5 533	0,9	119,5	5 008	27,0	0,6	7,8	35,3
Tigre	3	177	7 545	2,7	160,4	6 836,6	50,7	0,8	10,6	62,0
Tres de Febrero	4	57	2581	3,4	48,9	2 213,6	53,0	0,2	3,4	56,7
Vicente López	1	187	840	0,8	155,5	698,7	24,8	0,7	1,1	26,6
**Total**	**59**	**5 062**	**13 5108**	**52,9**	**4 555**	**120 685,9**	**1 053,2**	**21,6**	**187,0**	**1 261,8**

Cuadro de elaboración propia.

AVAD, años de vida ajustados por discapacidad; PBA, provincia de Buenos Aires.

**CUADRO 4:** Valor de los beneficios proyectados por obras de agua y alcantarillado al 2016

Beneficios estimados por mejoras a escenarios	Escenario I			Escenario II		
	Mínimo	Base	Máximo	Mínimo	Base	Máximo
Total en ARS^a^	1 079,4	3 262	8 952,3	891,4	2 701,2	7 456,3
Total en USD^b^	67,5	203,9	559,5	55,7	168,8	466,0

Cuadro de elaboración propia.

ARS, pesos argentinos; USD, dólares estadounidenses.

a Los valores de sepresan en millones de pesos argentinos en el año 2016.

b Los valores se expresan en millones de dólares estadounidenses en el año 2016 (cotización: 1 USD = 16 ARS).

## DISCUSIÓn

Los economistas suelen evaluar la mortalidad y morbilidad mediante la disponibilidad a pagar o el enfoque del capital humano, y les asignan un valor monetario a las muertes a través del valor presente del ingreso perdido por muerte prematura y el costo de la enfermedad por morbilidad (32). Los especialistas en salud traducen morbilidad y mortalidad a un número homogéneo y comparable (años vividos con discapacidad) mediante el cálculo de AVAD. Para comparar los AVAD evitados por la realización de obras de agua y saneamiento, con sus respectivos costos, se expresan en términos monetarios a través del salario (25).

Los beneficios así obtenidos ascienden a USD 203,9 millones al alcanzar el escenario I, con USD 559,5 millones el límite superior resultado del análisis de sensibilidad. Los mismos son bajos comparados con el costo de las obras, estimado en USD 10 289 millones a valores de 2016, alcanzan una cobertura de aproximadamente 95% en agua y 85% en saneamiento en el área considerada (33). Este resultado puede deberse, en primer lugar, a la subestimación de los datos de salud. La relación beneficio costo (B/C) es de 0,35 cuando se ajustan los beneficios por subestimación de los datos de salud.

Algunos estudios realizados a nivel internacional dan cuenta de una relación B/C de entre 1,4 y 4,8 para las intervenciones combinadas de agua y saneamiento de alto costo, con un valor de 4,7 para América Latina (34). No hay datos de intervenciones conjuntas publicados para Argentina, aunque, por ejemplo, para intervenciones de agua la relación B/C es de 2,5 (34). Gran parte de la diferencia se debe a la estimación de los costos de inversión de las obras. Otra parte se origina en la limitación del alcance de este trabajo. Al no incluir los beneficios generados por el valor del tiempo evitado (viajar o esperar para conseguir agua, tener que adaptar su calidad, hervirla, o acceder a una facilidad sanitaria), los cuales constituyen 80% de los beneficios totales de las obras según algunos autores (34).

Por otra parte, las siguientes limitaciones conllevan una subestimación de los beneficios generados por las obras de agua y saneamiento; no se consideran otras enfermedades; existe evidencia (35) de que la diarrea da cuenta de 38,6% de los AVAD estimados asociados al factor de riesgo de ASH inadecuados, también son importantes las enfermedades asociadas a la malnutrición (21%) y la malaria (14,2%); no se consideran los riesgos para la salud derivados de la concentración elevada de nitritos, nitratos y arsénico, sobre todo en las zonas donde se toma agua de pozo; no se incluyen beneficios sociales, como mejoras en los niveles educativos por mayor asistencia a la escuela, seguridad, privacidad, dignidad confort y estatus (22); y no se incluyen beneficios ambientales derivados de la conservación del recurso fluvial y de la reutilización de aguas residuales (el Río de la Plata es receptor de los desechos sanitarios que, sin tratamiento adecuado, encarecen el proceso de potabilización del agua extraída de él).

## CONCLUSIÓN

La importancia del ASH en la reducción de enfermedades es reconocida a nivel mundial. Si bien los beneficios calculados en este trabajo distan de alcanzar los costos que las obras implican, deben entenderse como un límite inferior de los potenciales beneficios que acarrean la realización de las obras. Mejoras en la información estadística disponible y la realización de estudios epidemiológicos locales, podrán permitir ir refinando los cálculos de este tipo en el futuro.

## Contribución de las autoras.

Ambas autoras concibieron el estudio original, recolectaron y analizaron los datos, eligieron el método para el análisis, interpretaron los resultados, escribieron y revisaron el manuscrito desde el primer borrador hasta versión final aprobada.

## Agradecimientos.

Las autoras agradecen a Agustín Shehadi su labor como ayudante de investigación.

## Financiación.

El trabajo que dio origen a este artículo se realizó en el marco de un proyecto de investigación de la Maestría en Evaluación de Proyectos de la Universidad del CEMA (Buenos Aires, Argentina).

## Declaración.

Las opiniones expresadas en este manuscrito son responsabilidad de los autores y no reflejan necesariamente los de la empresa AySA ni los de la Universidad del CEMA. Los datos en salud fueron todos obtenidos de organismos oficiales. Las opiniones expresadas en este manuscrito son responsabilidad del autor y no reflejan necesariamente los criterios ni la política de la *RPSP/PAJPH* y/o de la OPS.

## Footnotes

1 Dichos partidos corresponden al área de servicios de Agua y Saneamientos Argentinos S.A. (AySA) en Buenos Aires al momento de la realización de este trabajo. AySA es la empresa más grande en su tipo en Argentina.

4 A lo largo de todo el trabajo se considera un tipo de cambio promedio de 1 USD = 16 ARS para el año 2016.
